# Gut Microbiome-Based Therapeutics in Critically Ill Adult Patients—A Narrative Review

**DOI:** 10.3390/nu15224734

**Published:** 2023-11-09

**Authors:** Shiyue He, Fengyu Lin, Xinyue Hu, Pinhua Pan

**Affiliations:** 1 Center of Respiratory Medicine, Xiangya Hospital, Central South University, Changsha 410008, China; hsy0714@csu.edu.cn (S.H.); linfengyu@csu.edu.cn (F.L.); 2 FuRong Laboratory, Changsha 410078, China; 3 National Clinical Research Center for Geriatric Disorders, Xiangya Hospital, Changsha 410008, China; 4 Hunan Engineering Research Center for Intelligent Diagnosis and Treatment of Respiratory Disease, Changsha 410008, China

**Keywords:** gut microbiota, critically ill patients, fecal microbiota transplantation, short-chain fatty acid, gut microbiome-based therapeutics

## Abstract

The gut microbiota plays a crucial role in the human microenvironment. Dysbiosis of the gut microbiota is a common pathophysiological phenomenon in critically ill patients. Therefore, utilizing intestinal microbiota to prevent complications and improve the prognosis of critically ill patients is a possible therapeutic direction. The gut microbiome-based therapeutics approach focuses on improving intestinal microbiota homeostasis by modulating its diversity, or treating critical illness by altering the metabolites of intestinal microbiota. There is growing evidence that fecal microbiota transplantation (FMT), selective digestive decontamination (SDD), and microbiota-derived therapies are all effective treatments for critical illness. However, different treatments are appropriate for different conditions, and more evidence is needed to support the selection of optimal gut microbiota-related treatments for different diseases. This narrative review summarizes the curative effects and limitations of microbiome-based therapeutics in different critically ill adult patients, aiming to provide possible directions for gut microbiome-based therapeutics for critically ill patients such as ventilator-associated pneumonia, sepsis, acute respiratory distress syndrome, and COVID-19, etc.

## 1. Introduction

Intestinal dysbiosis, endotoxemia, and systemic inflammation are major factors contributing to pathophysiological alterations in critically ill patients. Critical illness is characterized by the loss of commensal microbiota and an excessive growth of potentially pathogenic bacteria, resulting in reduced production of short-chain fatty acids (SCFAs) or an inflammatory reaction induced by the gut microbiota [1], leading to prolonged immunosuppression and a high susceptibility to hospital-acquired infections [2,3]. In addition, prophylactic use of broad-spectrum antibiotics to treat infection is a common clinical approach, which aggravates the imbalance between the host immune system and the gut microbiota, suppressing the microbiota [4,5]. Thus, the ability of the original gut microbiota to prevent pathogen colonization is impaired, increasing the risk of infection [6,7]. In a critically ill mouse model, the upregulation of intestinal epithelial apoptosis and the enhancement of barrier hyperpermeability have been reported [8]. Moreover, an association between reduced gut microbiota diversity and increased relative abundance of potentially pathogenic bacteria, including aerobic gram-negative bacteria, has been found in several prospective cohort studies in critically ill patients with sepsis [9,10,11].

The intestinal microbiota inhibits the colonization of potential pathogens in the intestinal epithelium by stimulating the production of IgA, defensins, and antimicrobial peptides in the host, as well as competition for preferred resources among bacteria within specific ecological niches [12,13,14]. However, the increased colonization of pathogens and the disruption of the gut barrier can be affected by the collapse of microbiota diversity [14]. In addition, the intestinal microbiota can communicate with extraintestinal organs (e.g., lung, kidney, and heart, etc.) via bacterial extracellular vesicles [15]. Therefore, the repair of the gut microbiota and its metabolites may be suggested as a direction for the treatment or prevention of critical illness.

**Figure 1:**
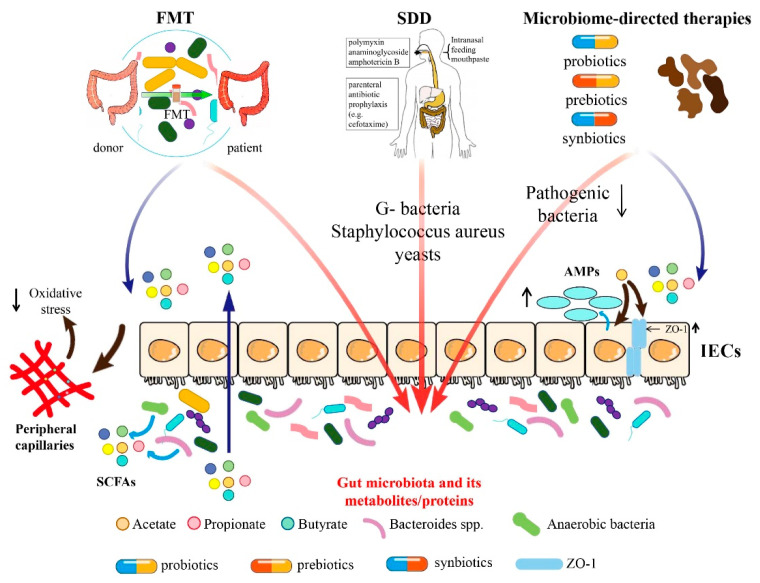
Summary of mechanism of different gut microbiome-based therapeutic approaches.

In this narrative review, we categorize the therapeutic approaches related to the gut microbiome into three groups: fecal microbiota transplantation (FMT), selective digestive decontamination (SDD), and microbiome-directed therapies including probiotics, prebiotics, synbiotics, and microbiota-derived metabolites and proteins (e.g., SCFAs and flavonoid) (Figure 1). We also summarize the application of different gut microbiome-based therapeutics in different critical illnesses, such as ventilator-associated pneumonia (VAP), sepsis, acute respiratory distress syndrome (ARDS), COVID-19, and other diseases (Table 1).

Gut microbiome-based therapies include FMT, SDD, supplementation of probiotics, prebiotics, synbiotics, and directly providing microbiota-associated metabolites and proteins. FMT plays a role by regulating gut microbiota and affecting SCFAs. Moreover, SDD mainly affects the colonization of G-bacteria, *Staphylococcus aureus*, and yeasts in the intestines. Microbiome-directed therapies play roles via decreasing pathogenic bacteria, increasing beneficial bacteria, and regulating the level of SCFAs. SCFAs could increase the expression of intestinal epithelial tight junction proteins and promote anti-microbial peptides (AMPs) secreted by intestinal epithelial cells (IECs), which move into peripheral capillaries and inhibit oxidative stress. FMT, fecal microbiota transplantation; SDD, selectively digestive decontamination; SCFAs, short-chain fatty acids; IECs, intestinal epithelial cells; and AMPs, anti-microbial peptides.

## 2. Methods/Data Search

The literature search included English-language articles published before September 2023 that belonged to Pubmed-index journals. We also searched the reference lists of the original papers for further relevant articles.

## 3. FMT

FMT is a therapeutic approach to transfer the micro-manipulated microbiota from healthy donor feces to the patient’s intestine, recovering intestinal diversity, inhibiting the growth of pathogenic bacterial communities in the gut environment, and driving competitive rejection of pathogenic bacteria among the local intestinal microbiota.

FMT reduces the levels of local or foreign bacterial pathogens in the intestinal microbiota of mice and restores the normal function of intestinal microbiota [48,49,50,51]. Early application of FMT reduces mortality in mice with myocardial infarction caused by chronic left anterior descending artery ligation [20]. It has been reported that FMT can alleviate lung inflammation and acute lung injury (ALI) in mice through modulating gut microbiota and metabolic disturbance [52]. In a sepsis mouse model, FMT can regulate the abundance of bacteria such as Firmicutes, Proteobacteria, *Escherichia Shigella*, and *Lactobacillus* to a level comparable to that of healthy mice, and downregulate the expression of the NOD-like receptor protein 3 (NLRP3) and Gasdermin-D (GSDMD)-N proteins and the release of inflammatory factors to inhibit cell pyroptosis [53]. Additionally, FMT restores immune homeostasis via providing specific colonies of bacteria which produce SCFAs (e.g., butyrate, etc.) with histone deacetylases inhibitory (HDACi) activity that can be amplified in inflammation and infection-induced systemic immune response, thereby restoring the interferon regulatory factor 3 (IRF3) signaling pathway [50]. In a mouse model of ALI, FMT restored the gut microbiota to reduce oxidative stress and impair the TLR4/NF-κB pathway in the lung, ameliorating lung damage [54]. In addition, in an ALI rat model, FMT could alleviate LPS-induced lung injury by modulating the TGF-β1/Smad/ERK pathway, and was able to regulate gut microbiota, inhibit immune-inflammation, and reduce inflammatory cytokines [55].

FMT application in healthy volunteers revealed a transient suppression of systemic immune cytotoxicity, with decreased T-cytotoxic CD8^+^ lymphocytes and natural killer cells in circulation, increased T-helper CD4^+^ cells, and an increased CD4^+^ to CD8^+^ ratio [56]. Clinical trials have demonstrated the ability of FMT in melanoma treatment. FMT can effectively shift the intestinal microbiota composition toward taxa favoring anti-programmed death-1 (PD-1) efficacy which is associated with increased intra-tumoral and peripheral antitumor immunity [57,58]. Moreover, FMT may act against antibiotic-resistant CDI [17,59] and contribute to recovery from immunotherapy for colitis and the eradication of colonized antibiotic-resistant bacteria in patients with hematological malignancies [18,60]. In addition, in a study of FMT for critically ill patients with antibiotic-associated diarrhea, good clinical outcomes were observed without infectious complications [61]. With the increasing importance of the microbiota in health, FMT treatment has been further investigated for application in autoimmune diseases, metabolic syndrome, sepsis, and other critical illnesses [62]. Moreover, clinical trials have shown that FMT applied to patients with severe alcoholic hepatitis presenting as acute-on-chronic liver failure (SAH-ACLF) rapidly improves the patients’ clinical severity scores, abnormal liver dysfunction and ascites, reduces pro-inflammatory cytokines levels, and is expected to significantly improve patient survival and the prognosis of hepatic encephalopathy [21]. Taken together, it can clearly be seen that FMT treatment may be a potential therapeutic option for critical illnesses.

## 4. SDD

For more than 30 years, SDD has been proposed as a measure to prevent infection in intensive care unit (ICU) patients, who mostly have respiratory failure, mechanism ventilation, reversible multiple organ failure, or are there after major surgery, a coma, or shock [63].Currently, SDD is only considered as a standard therapy in the Netherlands and is sporadically used in ICUs in other countries [64]. The main aim of SDD is to reduce ICU-acquired infections via eradicating and preventing colonization of the digestive tract by gram-negative bacteria, *Staphylococcus aureus*, and yeasts, thereby promoting the prognosis of ICU patients [65].

In a randomized controlled trial with 5939 enrolled patients, SDD has been demonstrated to be effective in reducing the mortality rate in ICU patients [66]. In addition, an SDD strategy in ICUs could reduce the clinically relevant infections caused by multidrug-resistant bacteria and decrease colistin- and tobramycin-resistant colonization with a nonsignificant, increasing rate of ICU colonization resistance [67]. A large, well-conducted, cluster, crossover, randomized trial showed that the hospital mortality of critically ill patients receiving mechanism ventilation with SDD was 27.0%, the hospital mortality of other patients without SDD was 29.1%. Although the result had no statistical significance, the authors conclude that the confidence interval includes a clinically relevant benefit [68]. Recently, a meta-analysis comparing the use of SDD with standard care in ICU patients, demonstrated that SDD contributed to a reduced risk of VAP, ICU-acquired bacteremia, and lower hospital mortality [69]. Furthermore, studies associating SDD strategy with COVID-19 have shown that SDD strategies have a beneficial effect on decreasing ICU mortality in mechanically ventilated patients with severe COVID-19 [22,23].

## 5. Microbiome-Directed Therapies

Loss of commensal microbiota and excessive growth of potentially pathogenic bacteria are the main features of the gut microbiota in critically ill adult patients [2]. Gut microbiota imbalance can increase the risk of secondary infection, immunosuppression, and even organ dysfunction, leading to an increased incidence of opportunistic infections and sepsis, aggravated various target organ damage, and worsened patient condition. Additionally, even after recovery from sepsis, the slow recolonization of patients’ normal microbiota may lead to long-term immunosuppression and poor prognosis. Therefore, different strategies related to the gut microbiota, such as using probiotics and prebiotics alone or in combination (synthetic preparations), have been proposed in order to prevent the further growth of pathogens and improve the outcomes of critically ill patients [25,70,71,72].

### 5.1. Probiotics

The International Scientific Association for Probiotics and Prebiotics defines probiotics as “live microorganisms that, when given in sufficient amounts, have a beneficial effect on the health of the host” [73]. They protect the intestinal barrier, attenuate pathogen overgrowth, decrease bacterial translocation, reduce serum pro-inflammatory cytokine concentrations while increasing the serum anti-inflammatory cytokine concentrations, and induce host immunomodulation to prevent infection [74,75,76,77]. In addition, probiotics also act through pharmacokinetics. For example, gut *Actinobacterium Eggerthella lenta* could affect the pharmacokinetic of digoxin and reduce its toxicity in treating congestive heart failure [78]. Moreover, the probiotic *E. coli* strain Nissle 1917 influences the pharmacokinetics of the antiarrhythmic amiodarone and increases drug absorption [79].

Several probiotics play a role in adult intensive care [80]. Probiotic therapy significantly reduces the incidence of diarrhea, acquired infections, and VAP in critically ill patients [24,26,81]. In sepsis-induced, severe ALI, *Akkermansia muciniphila* (*A. muciniphila*) was significantly negatively correlated with TNF-α, IL-1β and IL-6, suggesting that Gut *A. muciniphila* plays an important role in ALI and that supplementation with *A. muciniphila* may be a possible therapy for ALI [82]. In addition, the combination of probiotics *Bifidobacterium longum*, *Lactobacillus bulgaricus* and *Streptococcus thermophilus* was more effective as an adjuvant therapy for severe and critically ill patients with COVID-19, shortening the nucleic acid conversion time, and reducing the inflammatory index such as procalcitonin and C-reactive protein [83]. Another probiotic, *L. reuteri*., can reduce lung inflammation and mortality of ARDS [30]. In uremic dialysis patients, oral administration of Lactobacillus acidophilus led to a decrease in serum dimethylamine, a potential uremic toxin [84]. The administration of probiotics (e.g., *Bifidobacterium bifidum*, *Bifidobacterium catenulatum*, *Bifidobacterium longum*, *Lactobacillus plantarum*) can also significantly reduce serum proinflammatory endotoxin, decrease cytokine levels, and improve life quality [85]. A double-blind clinical study has shown that the probiotics *Lactobacillus rhamnosus* GG or a combination application of probiotics (including *streptococcus thermophiles*, *lactobacillus acidophilus*, *lactobacillus delbrueckii* ssp. *bulgaricus*, *lactobacillus paracasei*, *lactobacillus plantarum*, *Bifidobacterium longum*, *Bifidobacterium infantis*, and *Bifidobacterium breve*) has beneficial effects on heart failure caused by adverse cardiac remodeling after myocardial infarction (MI). These probiotics play roles through decreasing the levels of intestinal metabolite trimethylamine N-oxide (TMAO) [34,86].

### 5.2. Prebiotics

Prebiotics are substrates that can be selectively utilized by host microbes to maintain gut homeostasis and improve health outcomes. Dietary fiber (DF) is a powerful beneficial prebiotic that promotes the production of SCFAs [87].

Several prospective cohort studies have found that the use of DF in critically ill patients is effective in enhancing intestinal barrier function, reducing the systemic inflammatory response, modulating gut microbiota: increasing the abundance of the SCFAs-producing bacteria, and decreasing the level of potentially pathogenic microbiota [35]. The use of DF also improves the clinical outcomes, shortening hospital days, and reducing morbidity and mortality in critically ill patients [35]. Researchers have also found that prebiotics may ameliorate the prognosis of COVID-19 by offering anti-inflammatory nutrition, improving malnutrition, and enhancing immunity through the gut–lung microbial axis [36,37,88]. More importantly, DF-fermented SCFAs increase the production of CD103^+^DCs, promote the differentiation of activated CD8^+^T cells to effector cells with a memory phenotype, and improve the outcomes of anti-PD-1 immune checkpoint inhibitor therapy in patients with melanoma [89,90]. Similarly, the SCFAs produced by DF provide energy to the gut microbiota and promote the amino acids to reach the colon for absorption into the body rather than fermenting into uremic solutes [38].

In addition, flavonoid is an important class of natural products widely found in fruits which belongs to a class of plant secondary metabolites [91]. Clinical trials have demonstrated that flavonoids can upregulate the abundance of probiotics, such as *Bifidobaterium* and *Lactobacillus*, while downregulating the abundance of some pathogenic bacteria such as *Staphylococcus aureus* and *Clostridium histolyticum* [92,93]. Moreover, flavonoids can also promote the production of SCFAs [94].

### 5.3. Synbiotics

Synbiotics are mixtures of probiotics and prebiotics that exert beneficial effects on the host in two main ways: enhancing the viability of probiotic microorganisms and providing specific health effects [95,96]. Probiotics stimulated by prebiotics could regulate the metabolic activity of the gut, maintain the intestinal biostructure, and promote the growth and multiplication of probiotics and its resistance to reactive oxygen species and bile salts/acids [97]. In addition, synbiotic agents modulate the innate and adaptive immune systems to reduce systemic inflammation and promote extraintestinal organ function [71,97]. Synbiotics lead to lower concentrations of adverse metabolites which results in significantly increased SCFAs levels, which may contribute to a positive effect on the host health [95].

A double-blinded controlled clinical trial demonstrates that a combination of antibiotic intervention led to a significant reduction in pharyngeal aspiration in critically ill patients and an increased level of patient consciousness [98]. In addition, for hospital-acquired infections in critically ill patients, synbiotics may be a safer and more effective way to reduce endotoxin and inflammatory markers in serum and the complications of sepsis [76,81]. Studies have shown that prophylactic synbiotics (e.g., *Bifidobacterium breve* strain Yakult combined with *Lactobacillus casei* strain Shiorta, and galactooligosaccharides) increases the number of probiotics (e.g., *Bifidobacterium*, *Lactobacillus*) in fecal bacteria and intestinal SCFA levels, especially acetate. These may modulate the gut microbiota and environment, and have preventive effects on the incidence of enterocolitis and VAP in sepsis patients [25]. Moreover, synbiotics are used to maintain a stable intestinal microbiota after SIRS and major surgery including high-risk hepatectomy, colorectal resection surgery, Roux-en-Y gastric bypass (RYGB), and sepsis-associated encephalitis (SAE) [40,99]. Synbiotics could also reduce the incidence of diseases such as VAP and healthcare-associated pneumonia, and shorten ICU length of stay [100,101]. In the late stage of intestinal disorders, supplementation with synbiotics can accelerate the recovery of the microbiota, thus preventing the development of sepsis and the onset and progression of critical diseases such as ARDS to some extent [44]. Synbiotics intake has been demonstrated to reduce plasma levels of uremic toxin and may exert nephroprotective effects [84].

Based on the above literature, we briefly summarize the different scenarios in which FMT, SDD, probiotics, prebiotics, and synbiotics act directly by regulating the gut microbiota (Table 2).

### 5.4. Microbiota-Derived Metabolites and Proteins

Transfer of sterile filtrates from donor feces, rather than fecal microbiota, has been shown to be sufficient to restore normal bowel habits and eliminate symptoms [106]. Therefore, researchers speculate that gut metabolites may contribute to critical illness and dysbiosis. There is observational data that correlates critical illness, dysbiosis, and altered gut metabolites, including SCFAs, flavonoids, indole derivations, amines, bile acids, etc. [107]. In this section, several microbiota-derived metabolites and proteins therapies will be summarized.

#### 5.4.1. Short-Chain Fatty Acid

Typically, undigested DF, as well as proteins and peptides, can be fermented by gut bacteria in the cecum and colon. The main products of these fermentative reactions are SCFAs, consisting of groups of fatty acids with less than six carbons. SCFAs include formic acid (C1), acetic acid (C2), propionic acid (C3), butyric acid (C4), and valeric acid (C5). The major SCFAs in the gut are C2, C3, and C4, accounting for more than 95% of all SCFAs [108]. SCFAs are key mediators in the regulation of myocardial tissue repair by gut microbiota [20]. Decreased microbiota abundance has been shown to alter immune cell responses to infectious damage, usually resulting in a pro-inflammatory phenotype [109], which further aggravates disease progression. Normal levels of SCFAs support the activity of innate lymphocytes, T cells, and B cells in the gut, thereby improving immune tolerance in the gut, strengthening the gut immune barrier, and enhancing its ability to clear pathogens [14,110]. Furthermore, a large proportion of gut-derived SCFAs are transported out of the gut to affect other organs through the gut–lung axis, gut–brain axis, gut–liver axis, gut–kidney axis, gut–bone axis, gut–skin axis, gut–fat axis, gut–heart axis, and so on [111,112,113,114,115,116]. Available studies have shown that gut microbiota dysbiosis and a lack of SCAFs are significantly associated with the severity of COVID-19 [45]. Therefore, maintaining healthy gut microbiota and normal levels of SCFAs contribute to critical illness prevention and prognosis. However, little is known about their therapeutic mechanism in critically ill patients, except for mouse models, so verifying the therapeutic mechanism of SCFAs in clinical trials may be a possible direction in subsequent research. In the following sections, we introduce the beneficial role that major SCFAs play in critical illness.

##### Butyrate

Previous work on mouse models has shown that butyrate, a specific type of SCFAs readily produced from fiber-rich diets through microbial fermentation, is critical for the maintenance of intestinal homeostasis [117]. Thus, we speculate that butyrate acid plays a crucial role in critically ill patients.

In a mouse model of sepsis-associated encephalitis, butyrate is a major metabolite of intestinal microbiota and may have a neuroprotective effect in the process of sepsis through the GPR109A/Nrf2/HO-1 pathway [118].

Butyrate acid promotes the proliferation and differentiation of intestinal epithelial cells (IECs) and the synthesis of intestinal epithelial tight junction protein, such as the increased expression of Zo-1 and Occludin, reduces cell apoptosis, and inhibits intestinal permeability, resulting in enhanced intestinal mucosa mechanical barrier function [119,120]. In addition, butyrate acid enhances the intestinal mucosal immune barrier. It can maintain immune homeostasis by restoring the IRF3 signaling pathway [50]. Moreover, butyrate acid promotes the production of anti-microbial peptides (AMPs) [121]. AMPs are small molecular peptides produced by IECs with broad-spectrum antimicrobial activities, such as butyrate acid which promotes RegIIIγ and β-defensins in a GPR43-dependent manner, which play important roles in limiting bacteria and manipulating species composition [122,123]. Furthermore, butyrate acid strengthens the intestinal biological barrier function. Elevated levels of butyrate acid lowers colonic PH and inhibits the growth and colonization of pathogenic bacteria [124]. It also prevents bacterial translocation across the intestinal barrier by promoting the antimicrobial activity of intestinal macrophages [125].

As a histone deacetylase inhibitor, it affects the gene expression profile of intestinal epithelial cells and immune cells through histone deacetylases [126,127,128]. It can alter the gene expression profile of immune cells such as Treg cells, intestinal macrophages, and bone marrow-derived macrophages through G protein-coupled receptors (GPCR), affecting their response to microbial stimulation [125,129]. Butyrate acid can relieve inflammation and clinical symptoms in critically ill patients by activating Treg cells [130]. In addition to directly affecting the function of the intestine itself, butyrate acid can also affect the organs via the gut–lung axis and gut–brain axis. Studies have shown that the GPCR overlap with each other in extraintestinal or intestinal organs [131]. For example, GPR109A viewed as GPCR common to both lung and intestine can be activated by butyrate [108]. Butyrate acid affects the intestinal epithelial barrier function and immune function regulation through GPR109A, with similar effects on lung tissue (Table 3). Intraperitoneal injection of sodium butyrate acid can decrease the expression of hypermobility protein 1 (HP-1), pro-inflammatory cytokines such as tumor necrosis factor-α (TNF-α) and interleukin-6 (IL-6), and inhibit the activation of the NF-κB signaling pathway in an ALI/ARDS mouse model [60,132].

##### Propionate

Propionate is mainly produced by Bacteroides spp. and is used as a gluconeogenic substrate in the liver and intestinal to provide energy to the body [124]. Propionic acid is an anti-inflammatory cytokine, its level in serum may predict the severity and prognosis in critically ill patients and may be a cytokine regulatory marker for critical illnesses such as sepsis [144].

##### Acetate

Acetate is mainly derived from gut microbes and is a metabolite released into the intestinal lumen by anaerobic bacteria from the gut microbiota, which is then absorbed by IEC and distributed to peripheral capillaries [145]. In recent years, studies in mice have found that acetate inhibits the permeability of the alveolar–capillary barrier, reduces pulmonary edema, inhibits oxidative stress, suppresses inflammatory cell recruitment and inflammatory mediator production, and regulates MAPK pathway activation; thus leading to amelioration of ALI and ARDS [146]. Elevated levels of acetate may prevent IEC translocation by inhibiting endotoxin and increasing claudin, thereby reducing the incidence of sepsis [25,71,147].

#### 5.4.2. Flavonoid Metabolites

Researchers have found that flavonoids ingested with food enter the circulatory system through the intestines to exert their beneficial effects [148]. Generally, flavonoids are absorbed as metabolites, and gut microbiota participate in this metabolism [149,150]. Flavonoid metabolites can shape the gut microbiota by inhibiting the growth of various pathogens and increasing beneficial genera; these could reduce the endotoxin, maintain gut immune homeostasis, and promote nutrients absorption [151,152]. Flavonoids metabolites have anti-inflammatory effects and play a part in local and systemic immunity. In mouse models, flavonoid metabolites can improve intestinal barrier function by reducing intestinal mucosal inflammation and maintaining the intestinal tight junction barrier and structure [46,153,154]. In addition, flavonoid metabolites regulate inflammatory mediators, such as through inhibiting endothelial activation, NLRP3 inflammasome, toll-like receptors (TLRs), or bromodomain-containing protein 4 (BRD4), as well as activating nuclear factor erythroid-derived 2-related factor 2 (Nrf2), thereby restoring cytokine storm in critical illness including SARS-CoV-2 infection [47]. DAT, a kind of flavonoid metabolite, has been shown to treat upper respiratory tract infections in mice and protect mice from bacterial endotoxin-induced septic shock [155,156]. Therefore, the researchers suggest that flavonoid metabolites produced by intestinal microbial metabolism play important roles when critically ill patients and hosts are infected with viruses and lethal bacterial infections.

#### 5.4.3. Others

Other than SCFAs and flavonoid metabolites, other microbiota-derived metabolites and proteins also play a critical role in maintaining the balance of intestinal mucosa and contributing to the treatment of critical illness. Indole-3-propionic acid (IPA) could modulate gut microbiota in normal mice, increase the levels of some probiotics (e.g., Akkermansiaceae, Bifidobacteriaceae), strengthen the mucus barrier, and attenuate LPS-induced inflammatory factors in sepsis by increasing mucins and goblet cell secretion products [157]. Studies on mouse models of sepsis suggest that the anti-inflammatory activity of IPA may associated with the increased abundance of Bifidobacteriaceae and inhibited expansion of Enterobacteriaceae, contributing to an improvement in mortality in sepsis [158]. Aromatic microbial metabolites (AMMs), such as phenyl lactic and 4-hydroxyphenyllactic acids, have been observed at a much higher level than normal in the serum of septic patients [159]. A prospective observational pilot study found that high level of AMMs were associated with severity and mortality in critically ill patients, and may become a possible direction to improve the prognosis of critical illness [160]. TMAO has been found to be higher in individuals with heart failure than in controls, suggesting that TMAO is a new and novel risk factor in heart failure development and can lead to cardiac hypertrophy and cardiac fibrosis [161]. Beta-lactamase, produced primarily by extended-spectrum β-lactam Enterobacteriaceae, has been shown to reduce the jejunal concentration of antibiotics and to prevent antibiotics from reaching the colon, thus alleviating the effect of antibiotics on gut microbiota disturbance [162,163].

## 6. Limitations

**Figure 2:**
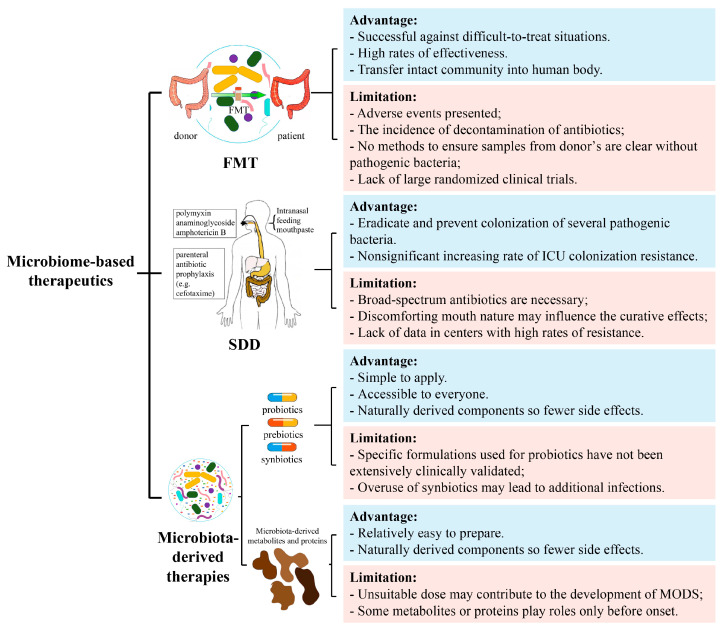
Advantages and limitations of gut microbiome-based therapeutics. Including FMT, SDD, and microbiota-derived therapies. FMT, fecal microbiota transplantation; SDD, selectively digestive decontamination; MODS, multiple organ dysfunction syndrome.

The gut microbiome has been recognized as a potential tool for the prevention and treatment of critical illness. However, although different microbiome-based therapeutics have proven to be effective in the treatment of critical illness, there remain limitations and challenges for future development (Figure 2).

### 6.1. Limitations of FMT

Adverse events in FMT include excessive flatulence, reflux, and the requirement of discontinuation of antibiotics in patients, which greatly increase complications such as *E. coli* bacteremia, lactobacillus bacteremia, and bacterial peritonitis [62,164,165]. More importantly, it is not entirely clear which bacteria are inhibited after FMT, and there is no suitable method to screen for potentially pathogenic bacteria in donor samples [14]. In addition, the lack of large randomized clinical trials remains a non-negligible limitation [103].

### 6.2. Limitations of SDD

Although SDD has been reported as an effective strategy to reduce ICU inpatient mortality, there are still some barriers to its widespread clinical use. One of the barriers is the concern that the widespread use of broad-spectrum antibiotics might promote antimicrobial-resistant organisms [166]. Additionally, it has been noted that, in patients on prolonged ventilation in the ICU, dosing procedures can be optimized due to the discomforting nature of oral creams and the reduced access of gastric suspensions to the upper gastrointestinal tract, which may bias in clinical trials [68]. Indeed, little data is available in centers with high rates of resistance and without long-term follow-ups, and the effect of SDD on the incidence of antimicrobial-resistant organisms is still unsolved [69]. Further studies will also need to assess the impact on secondary resistance and disruption patterns in the gut microbiome.

### 6.3. Limitations of Probiotics, Prebiotics and Synbiotics Therapies

Not all studies have shown optimistic results for microbiome-directed therapies [165,167,168], and there are also several risk factors in critical illness. On the one hand, the current clinical trial results of microbiota-directed therapy do not fully support their preventive role in critically ill patients [168,169,170]. The suitable probiotics for each dysbiosis situation are difficult to find and using them alone reduces their efficacy. Thus, combinations with other components are needed, but the specific formulation used for probiotics have not been extensively clinically validated [171]. On the other hand, overuse of synbiotics in microbiome-directed therapy not only fails to treat nosocomial infections in critically ill patients, but also leads to additional infectious complications [75].

### 6.4. Limitations of Intestinal Microbial Metabolite Therapy

High levels of SCFAs may have direct cytotoxic effects on pathogens and contribute to the development of MODS [144,172]. Moreover, after rectal administration or oral administration of butyrate acid, the proportion of Bacteroidetes phylum increases but the proportion of the thick-walled phylum decreases, which is detrimental to the restoration of gut microbiota homeostasis in critically ill patients [173,174]. In a variety of diseases, such as influenza, DAT treatment activates the immune system; however, it was only effective when DAT was given before the onset of infection. If it was given after the infection, it may exacerbate the progression of the disease [155].

## 7. Conclusions and Further Directions

FMT and SDD, as well as probiotics, prebiotics, and synbiotics, can restore the original intestinal microecology in critically ill patients, reduce the inflammatory response, and decrease the incidence of infectious complications. Gut microbial metabolites may improve the clinical condition of critically ill patients by enhancing immune tolerance and alleviating inflammatory response. Therefore, gut microbiome-based therapeutics may be applicable to critically ill adult patients.

Although current evidence suggests that gut microbiome-based therapeutics are beneficial for critically ill adult patients, there are still some issues that need to be validated in further studies in human and mouse models to continue exploring mechanisms. In our view, firstly, the toxicity and appropriate therapeutic doses of probiotics, prebiotics, synbiotics, and gut microbiota metabolites should be evaluated. Second, the appropriate composition of FMT grafts should be determined to ensure patient safety. Third, more clinical trials should be conducted for more types of critical illness, and the number of patients or cases needs to be expanded. Fourth, further molecular mechanisms of action need to be explored, which may contribute to the new therapy targets. Finally, the application of gut microbiome-based therapeutics for prevention of critical illness may be more desirable than the treatment of critical illness.

In addition to deeper discussions about the mechanisms and studies that need to be validated by mouse models, further studies of new and useful materials, technologies, and methodologies, such as lactomodulin, symbiotic microbial consortia, and engineered symbiotic bacteria, may also be a direction in gut microbiome-based therapeutics of critical illness [175].

## Figures and Tables

**Table 1:** The application of gut microbiome-based therapeutics in critically ill adult patients.

The Gut Mcirobiome-Based Therapeutics	Disease
FMT	Critically ill patients without CDI [16], antibiotic-resistant bacteria [17,18], CDI [19], MI [20], SAH-ACLF [21]
SDD	VAP [22,23]
Probiotics	Diarrhea and acquired infections in critically ill patients [24,25], VAP [26,27], COVID-19 [28,29], ARDS [30,31,32], uremia [33], heart failure [34]
Prebiotics	Sepsis [35], COVID-19 [36,37], uremia [38]
Synbiotics	Sepsis [39], systemic inflammatory response syndrome (SIRS) [40], high-risk hepatectomy and major surgeries [41], uremia [42], VAP [43], ARDS [44]
SCFAs	COVID-19 [45], MI [20]
Flavonoid	Sepsis [46], COVID-19 [47]

CDI, clostridium difficile infection; MI, myocardial infarction; SAH-ACLF, severe alcoholic hepatitis presenting as acute on chronic liver failure; VAP, ventilator-associated pneumonia; ARDS, acute respiratory distress syndrome; SIRS, systemic inflammatory response syndrome.

**Table 2:** Appropriate situations for different therapeutic approaches.

Mirobiome-Based Therapeutics	Appropriate Situations	References
FMT	Post-antibiotics; CDI; the possible eradication of colonization and recurrent infections due to different species of MDROs.	[102,103,104]
SDD	Wards with low rates of resistant bacteria; patients colonized with Staphylococcus aureus or aerobic gram-negative bacteria.	[103,105]
Probiotics, prebiotics and synbiotics	Available as food supplements; most situations with gut microbiota dysbiosis except post-antibiotics.	[73,102]

FMT, fecal microbiota transplantation, SDD, selectively digestive decontamination, CDI, Clostridium difficile infection, MDROs, multidrug-resistant organisms.

**Table 3:** Association of G protein-coupled receptor with SCFAs.

GPCR	Association with SCFAs	References
GPR41	Activated by SCFAs such as propionate, butyrate and valerate. SCFAs enhance the cellular metabolism and adaptive immunity function through GPR41 and maintain intestinal homeostasis. Propionate activated GPR41 alleviates inflammation in allergy.	[133,134,135]
GPR43	GPR43 can be activated by SCFAs such as acetate, propionate, butyrate. SCFAs promote intestinal epithelial cells to produce AMPs, stimulates the migration of neutrophils, and modulates the production of reactive oxygen species (ROS) and phagocytosis through a GPR43-relate manner. Elevated SCFAs level enhances luminal immunoglobulin A production and strengthen the intestinal mucosa barrier.	[123,136,137,138,139,140]
GPR40	GPR40 receptor activation promotes tight junction assembly in airway epithelial cells via AMPK-dependent mechanisms.	[141]
GPR109A	Butyrate-mediated GPR109A activation suppresses inflammation and regulates lipid metabolism by regulating macrophages.	[108,142,143]

GPCR/GPR, G protein-coupled receptor; SCFA, short-chain fatty acid; AMP, anti-microbiota peptide.

## Data Availability

Not applicable.
